# Biomarkers of Rehabilitation Therapy Vary according to Stroke Severity

**DOI:** 10.1155/2018/9867196

**Published:** 2018-03-12

**Authors:** Erin Burke Quinlan, Lucy Dodakian, Jill See, Alison McKenzie, Jill Campbell Stewart, Steven C. Cramer

**Affiliations:** ^1^Department of Anatomy & Neurobiology, University of California, Irvine, CA, USA; ^2^Department of Neurology, University of California, Irvine, CA, USA; ^3^Department of Physical Therapy, Chapman University, Orange, CA, USA; ^4^Department of Exercise Science, University of South Carolina, Columbia, SC, USA

## Abstract

Biomarkers that capture treatment effects could improve the precision of clinical decision making for restorative therapies. We examined the performance of candidate structural, functional, and angiogenesis-related MRI biomarkers before and after a 3-week course of standardized robotic therapy in 18 patients with chronic stroke and hypothesized that results vary significantly according to stroke severity. Patients were 4.1 ± 1 months poststroke, with baseline arm Fugl-Meyer scores of 20–60. When all patients were examined together, no imaging measure changed over time in a manner that correlated with treatment-induced motor gains. However, when also considering the interaction with baseline motor status, treatment-induced motor gains were significantly related to change in three functional connectivity measures: ipsilesional motor cortex connectivity with (1) contralesional motor cortex (*p* = 0.003), (2) contralesional dorsal premotor cortex (*p* = 0.005), and (3) ipsilesional dorsal premotor cortex (*p* = 0.004). In more impaired patients, larger treatment gains were associated with greater *increases* in functional connectivity, whereas in less impaired patients larger treatment gains were associated with greater *decreases* in functional connectivity. Functional connectivity measures performed best as biomarkers of treatment effects after stroke. The relationship between changes in functional connectivity and treatment gains varied according to baseline stroke severity. Biomarkers of restorative therapy effects are not one-size-fits-all after stroke.

## 1. Introduction

Restorative therapies that promote plasticity within surviving neural tissue [1] can improve recovery, but patient responses are highly variable. Identifying biomarkers that provide information about neural events underlying treatment-related gains could improve individualization of rehabilitation therapy [2], as stroke rehabilitative care clinical decision making is still primarily based on behavioral assessments [3].

A biomarker can be defined as a laboratory measurement reflecting the activity of a disease process [4] that changes in parallel with clinical status [5]. Imaging techniques have been identified that are related to neural events underlying brain plasticity [6, 7] and are candidate biomarkers of treatment-induced motor gains after stroke [8–10], including functional MRI (fMRI) [11] and diffusion tensor imaging (DTI) [7, 12, 13]. Restorative therapies in stroke animal models have been associated with induction of angiogenesis derived from T2^∗^-weighted susceptibility-weighted imaging (SWI) [14], but this MRI-based measure has been little studied in humans as a potential biomarker related to plasticity.

The current study compared the utility of these imaging-based biomarker candidates based on the extent to which each changed in parallel with motor gains across a course of therapy in patients early in the chronic phase of stroke. The first study hypothesis was that motor gains across three weeks of standardized motor therapy would correlate significantly with changes in measures of cortical activation, functional connectivity, and corticospinal tract (CST) integrity; changes in angiogenesis were also examined as an exploratory aim. These categories of brain imaging have not previously been directly compared. Prior reports have emphasized the biomarker potential of activation [15, 16] and connectivity measures [17–19] in terms of detecting changes in brain function that parallel treatment-derived behavioral gains in the chronic phase poststroke, and so these two categories of brain imaging measures were of relatively high interest to this hypothesis; connectivity measures were of particular interest because they provide insights into network interactions rather than activity in a single network node [20]. Because of the high heterogeneity of human stroke, a single biomarker may not perform in an identical manner across different patient subgroups [21]. A second hypothesis, therefore, was that the relationship between motor gains and change in biomarker values over time would vary significantly according to baseline motor status.

## 2. Materials and Methods

### 2.1. Patients

Patients enrolled in a study of robotic therapy (ID number NCT01244243) underwent a battery of assessments twice, baseline (“pretreatment”), then again immediately upon completion of treatment (“posttreatment”). Inclusion and exclusion criteria (Table 1) were focused on capturing patients who were close to the time when spontaneous motor recovery is complete and who had stable motor deficits. To confirm patients had reached a plateau in arm motor recovery, the arm motor Fugl-Meyer (FM) scale [22] was performed at two baseline exams separated by a week, and patients could only advance if the two scores did not differ by more than 3 points. Biomarker testing was obtained in 31 consecutive enrollees and is the focus of the current report; of these, 13 were excluded (see the bottom of Table 1 for specific reasons), leaving 18 patients who are the focus of the current report. These 18 patients (Table 2) were 61 ± 10 years of age (mean ± SD) and 4.1 ± 1 months poststroke, and all but two had ischemic injury. Enrollees showed wide variation in baseline clinical status, for example, FM scores ranged from 20 to 60 (66 = best). All patients provided written informed consent. The UC Irvine Institutional Review Board approved this study.

### 2.2. Robotic Therapy

Patients underwent 12 two-hour treatment sessions of robotic hand therapy over a three-week period. All 12 robotic therapy sessions were completed by 100% of patients; this was achieved by organizing treatment time schedules to accommodate patient needs. The distal arm on the patient's paretic side was secured to the robotic device. Therapy consisted of repeated grasp-release (“close” and “open”) movements of the affected hand/wrist, linked to a range of games and exercises, using a pneumatically actuated robotic device described previously [23]; the patient attempted movement on cue, and the robot moved the patient's hand after a delay, if movement was incomplete. All therapy sessions were directly managed by a licensed occupational therapist or physical therapist at the patient's side at all times. The average number of movement repetitions at each of the 12 sessions was 954 for the fingers, 2579 for the thumb, and 1298 for the wrist.

### 2.3. Image Acquisition

An MRI scan was performed at baseline and again approximately one week posttreatment. MRI images were acquired using a 3.0T Philips Achieva system. Three runs of blood oxygenation level-dependent (BOLD) images were acquired using a T2^∗^-weighted gradient-echo planar imaging sequence (TR = 2000 ms, TE = 30 ms, 31 slices with thickness 4 mm and 1 mm interslice gap). Data were acquired using a block design while patients were visually guided to alternate between 24-second blocks of rest versus execution of paretic hand grasp-release movements (0.125 Hz movement cycle), emulating the content of robotic training, while wearing a nonactuated plastic exoskeleton identical to the robotic interface. Each of the three fMRI runs acquired 48 brain volumes, contained two blocks each of rest and movement, and spanned 96 seconds. Before the MRI, patients were trained to achieve successful, independent performance on the task. Throughout the fMRI scan, patients were visually monitored, and an investigator evaluated task performance. Anatomical imaging included a high-resolution T1-weighted image using a 3D MPRAGE sequence (TR = 8.5 ms, TE = 3.9 ms, slices = 150, voxel size = 1 × 1 × 1 mm^3^, acquisition time = 9.58 minutes), diffusion tensor imaging (32 directions, *b* value = 1000 smm^−2^, 60 slices, voxel size = 1.75 × 1.75 × 2 mm^3^, acquisition time = 6.6 minutes), and susceptibility-weighted imaging (TR = 35 ms, TE = 20 ms, slices = 128, voxel size = 0.72 × 0.72 × 1 mm^3^, acquisition time = 3.73 minutes).

### 2.4. Data Analysis

#### 2.4.1. Treatment-Induced Motor Gains

The primary outcome measure of the study was arm motor gains from robotic therapy, defined by combining impairment-based (FM) [22] and function-based (Action Research Arm Test (ARAT)) [24] assessments via principal component analysis [25, 26]; only the first component was used given that it accounted for 74% of variance. Treatment-induced motor gains were measured as the change from baseline to immediately posttreatment and were examined in relation to the below candidate biomarkers (Table 3).

#### 2.4.2. Functional MRI

The fMRI images were analyzed using SPM8 (Wellcome Trust Centre for Neuroimaging, London, UK). Functional data were processed as previously described [27]. Primary motor cortex (M1) and dorsal premotor cortex (PMd) in each hemisphere were the chosen regions of interest (ROI), as they were hypothesized to be of greatest importance to biomarker assessment. For the move versus rest contrast, peak beta contrast estimates and activation volumes were extracted from each ROI using small volume correction using threshold *p* < 0.001.

#### 2.4.3. Functional Connectivity

A psychophysiological interaction (PPI) analysis was used to assess functional connectivity. Psychophysiological interaction analysis is a tool that can evaluate the influence of specific variables (here, pre- versus posttreatment scan) on physiological variables (BOLD response) [28, 29] and that reflects underlying neuronal changes [30]. For each patient, time series data were extracted from the BOLD fMRI images, for each of the four ROIs. The PPI design matrix contained the following 3 regressors: (1) the psychological variable (i.e., pre- versus posttreatment session); (2) the physiological variable (i.e., ROI BOLD time series data); and (3) the interaction term of these two variables (i.e., the PPI term). Changes in motor network functional connectivity were evaluated as the changes in PPI regression slopes between brain regions based on pre- versus posttreatment scan. Specifically, to evaluate the change in functional connectivity between two brain regions, the time series data from one ROI was plotted against the time series of a second ROI, for both the pre- and the posttreatment fMRI sessions. Next, the slopes of the regression lines for the two fMRI sessions were extracted [31] and the difference in slope across treatment (post- minus pretreatment) was calculated. An increase in the PPI slope from pre- to posttherapy indicates that activity between the two brain regions has become more correlated, and thus functional connectivity increased; a decrease in the PPI slope over time indicates decreased functional connectivity. In this way, connectivity was measured between ipsilesional M1 (iM1) and (1) contralesional M1 (cM1), (2) ipsilesional PMd (iPMd), and (3) contralesional PMd (cPMd). Adopting a control measure employed in preclinical stroke studies [32], functional connectivity was also evaluated between two nonmotor regions, ipsilesional and contralesional primary visual cortexes (V1).

#### 2.4.4. Corticospinal Tract Integrity

CST white matter integrity was measured using diffusion-weighted images, as previously [27]. The ipsilesional cerebral peduncle was selected for assessing CST integrity, via fractional anisotropy (FA), because corticospinal tract fibers are highly focused in this region.

#### 2.4.5. Peri-Infarct Angiogenesis

Serial T2^∗^-weighted SWI scans were used to estimate angiogenesis across treatment [14]. Two infarct rims were generated in native SWI space, as per a prior report [33]. First, the infarct was outlined on the baseline T1-weighted images. Using FSL (http://www.fmrib.ox.ac.uk/fsl), each lesion mask was dilated twice, by 1 mm each time. Rim 1 was defined as the first dilatation minus the infarct, while Rim 2 was defined as the second dilatation minus infarct + Rim 1. For posttreatment, the patients' lesion masks (drawn in pretreatment SWI space) were transformed into posttreatment SWI space, then the two posttreatment Rims were created. To ensure Rims did not extend beyond the brain, the FAST module in FSL was used to generate pre- and posttreatment CSF masks that were then subtracted from each infarct Rim. The mean intensity within each Rim was calculated, at both time points, to calculate the change in SWI signal intensity from pretreatment to posttreatment.

#### 2.4.6. Statistics

To address the first hypothesis that the candidate biomarker would change in parallel with motor gains, linear regression was used to compare “change in biomarker,” from pretherapy to posttherapy for each candidate measure, with “treatment-induced motor gains” over the same time period, that is, from pretherapy to immediately posttreatment. To address the second hypothesis that the relationship between biomarker change and motor gains would vary according to the patient subgroup, patients were divided into two “baseline impairment subgroups” based on a median split of baseline impairment (pretherapy FM scores); this turned out to be baseline FM ≤ 36 for the more impaired subgroup versus FM > 36 for the less impaired subgroup, a division that closely parallels FM severity subgrouping based on cluster analysis [34]. The above linear regression analyses to predict “treatment-induced motor gains” were then repeated but with the inclusion of the interaction term “change in biomarker X baseline impairment subgroup” in each model. For any model in which the interaction term was significant, post hoc testing was done separately for each baseline impairment subgroup. Analysis of fMRI and PPI data excluded any patient in whom substantial (>50%) damage was present in any of the ROIs [19, 35]; this occurred in three patients from the more impaired subgroup and in two patients from the less impaired subgroup. Bonferroni correction was applied for multiple comparisons: three for functional connectivity and so a *p* value of 0.0167 was used to define significance; eight for regional activation and so a *p* value of 0.00625 was used to define significance; and no correction was needed for SWI or DTI, as for each there was only a single candidate measure (Table 3). Parametric statistical methods were used for measures that were normally distributed or could be transformed to a normal distribution; otherwise, nonparametric methods were used. All analyses were two-tailed with alpha = 0.05 and used JMP-8 software (SAS).

## 3. Results

### 3.1. Patients

Patients showed wide variation in treatment-related clinical improvement; for example, gain in FM scores from baseline to the end of treatment was 2.9 ± 2.7 points (*p* = 0.0003), with 25% of patients achieving clinically relevant gains of 6 points or more. During fMRI scanning, all subjects had at least some hand movements, with a full range of motion present in 5/9 subjects in the less impaired group and 1/9 in the more impaired group (*p* = 0.13, Fisher's exact test). Mirror movements in the unaffected hand during intended movement of the affected hand during fMRI were present in 1/9 persons in the less impaired group and 2/9 persons in the more impaired group (*p* = 1.0, Fisher's exact test).

Regarding the candidate biomarker measures (Table 3), none changed significantly over time; the performance of each as a biomarker was then evaluated by comparing its change over time with the extent of treatment-induced motor gains.

### 3.2. Biomarker Performance: All Patients Combined

Biomarker performance across all patients was evaluated by examining the extent to which change in each assessment over time paralleled treatment-induced motor gains. When evaluating the candidate measures among all patients combined, no biomarker candidate changed over time in parallel with motor gains. One measure, degree of increase in iM1-cM1 connectivity, showed a trend towards correlating with larger motor gains (*r* = 0.53, *p* = 0.065).

### 3.3. Biomarker Performance as a Function of Degree of Baseline Deficits

**Figure 1:**
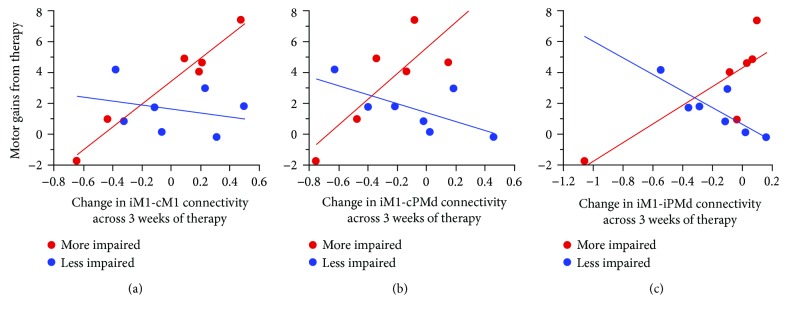
Biomarker performance varies according to baseline motor deficits. Changes in functional connectivity paralleled treatment-related behavioral gains in a manner that varied according to severity of pretreatment impairments. The change in connectivity between ipsilesional primary motor cortex (iM1) and (a) contralesional M1 (cM1), (b) contralesional PMd (cPMd), and (c) ipsilesional dorsal premotor cortex (iPMd) are each plotted against motor gains from therapy. Motor gains were assessed as the principal component of the change in Fugl-Meyer (FM) and Action Research Arm Test scores from baseline to immediately posttherapy; to aid interpretation, change in FM score is presented on the *y*-axis. Baseline motor impairment was split amongst all enrollees based on median FM score at baseline, with more impaired patients (red dots) having FM ≥ 36, and less impaired patients (blue dots) having FM < 36. A significant interaction term (change in connectivity over time × baseline impairment subgroup) was identified for all three measures of functional connectivity (iM1-cM1: *p* = 0.003; iM1-cPMd: *p* = 0.005; iM1-iPMd: *p* = 0.004).

The relationship that change in each assessment over time had with treatment-induced motor gains was further examined as a function of baseline motor impairment. For all three measures of motor system functional connectivity examined, the interaction term (change in connectivity over time × the baseline impairment subgroup) was significant (Figure 1) and survived Bonferroni correction (*p* < 0.0167): iM1-cM1 (*p* = 0.003), iM1-cPMd (*p* = 0.005), and iM1-iPMd (*p* = 0.004). Note that connectivity at baseline did not significantly differ between the two subgroups (iM1-cM1: *p* = 0.27; iM1-cPMd: *p* = 0.76; and iM1-iPMd: *p* = 0.20). Also, the control measure was negative, as the interaction term between change in iV1-cV1 connectivity and baseline impairment subgroup was not significant (*p* = 0.19). For the eight measures of regional activation predicting motor gains, none was significant after Bonferroni correction (*p* < 0.00625). The interaction term was not significant for the measurements related to CST integrity or peri-infarct angiogenesis.

For the candidate biomarker measures showing significant interaction terms, post hoc analysis (Fisher's Least Significant Difference test) was then performed separately for each of the two baseline impairment subgroups. See Table 2 for the characteristics of the two subgroups; note that apart from the FM score, which was the defining basis for separating the two subgroups, there were no differences between subgroups including with respect to age, total infarct volume, NIHSS total score, or CST integrity. In the *more impaired subgroup* (baseline FM ≤ 36), where three patients with substantial (>50% [19, 35]) damage to an ROI were excluded, *larger* treatment-induced motor gains were associated with significant *increases* in functional connectivity, between iM1 and cM1 (*r* = 0.98, *p* = 0.0007; Figure 1(a)), iM1-cPMd (*r* = 0.82, *p* = 0.046; Figure 1(b)), and iM1-iPMd (*r* = 0.83, *p* = 0.04; Figure 1(c)). In the *less impaired subgroup* (baseline FM > 36), where two patients with substantial damage to an ROI were excluded, *larger* treatment-induced motor gains were associated with significant *decreases* in functional connectivity, between iM1 and cPMd (*r* = −0.83, *p* = 0.02; Figure 1(c)), with a trend for decreased iM1-cPMd connectivity (*r* = −0.68, *p* = 0.09; Figure 1(b)).

## 4. Discussion

Stroke is a very heterogeneous disease, complicating prescription of restorative therapies. Biomarkers reflect disease-related events underlying behavioral state and its evolution [4, 5] and therefore may be useful to inform treatment decisions in this context. Numerous candidate biomarkers have been suggested, but to date, results across studies show substantial variability. The current study compared the performance of several imaging-based candidate measures previously suggested as useful biomarkers and, in addition, considered how results varied according to the level of baseline motor deficits. Across the entire (and heterogeneous) stroke population studied, no biomarker changed significantly in parallel with treatment-induced motor gains. However, significant findings emerged when biomarkers were examined in specific patient subgroups, with the choice of biomarker and the direction of its relationship with treatment-related behavioral gains varying according to the severity of baseline motor impairment. Of the various imaging candidates examined, measures of functional connectivity performed best as biomarkers.

Functional connectivity measures performed best as biomarkers in this chronic stroke population, and importantly, this was only true when examining patient subgroups defined according to baseline deficits. Previous studies have emphasized the importance of such connectivity measures for understanding recovery after stroke [36]. The functional connectivity findings were specific to the motor system given that changes in iV1-cV1 connectivity did not significantly correlate with treatment gains, similar to findings in an animal stroke model [32] and a prior study of patients with stroke [37]. Regional measures of cortical activation were considerably weaker biomarkers of treatment-induced motor gains, suggesting a network-level approach is more informative than such regional measures for understanding behavioral changes that parallel motor training. The change in the DTI measure of CST integrity was also not related to behavioral change perhaps because induction of white matter changes may require far more extensive intervention [12]. Change in the peri-infarct SWI, a variable not previously studied as a biomarker of brain plasticity in humans after stroke, also was not significant, suggesting that this MRI measure, validated in rodents during the early weeks poststroke [14], may not be useful in humans, at least across a three-week intervention during the chronic stage of stroke.

Across the entire population of enrollees, no biomarker changed over time in a manner that correlated significantly with treatment-induced motor gains. Mixed results have been seen in prior studies examining changes in connectivity as a biomarker of restorative therapy effects after stroke, with increased [17] and decreased [38] connectivity over time reported as correlating with treatment gains. These disparities may be due to differences in the study design such as the method used to measure connectivity, choice of treatment, or severity of deficits among enrollees. Another key factor is the extent of treatment-induced behavioral gains. Gains in the current study on average were modest in magnitude, and findings might have differed if larger gains had been achieved. Further insight was obtained in the current study when biomarker performance was examined within specific patient subgroups. This approach was motivated by the frequent finding that features of spontaneous [39] and treatment-induced [22] recovery vary tremendously according to the level of baseline behavioral deficits. Consistent with this and in support of the second study hypothesis, current results differed substantially according to the level of baseline deficits (Figure 1).

Among patients in the *more impaired subgroup*, larger treatment-induced motor gains were associated with *increased* motor system functional connectivity over time in all instances, particularly between iM1 and cM1. This is consistent with prior findings: increased activity within contralesional brain regions is associated with greater motor impairment after stroke [25], larger facilitatory effect of cPMd on iM1 is seen in patients with greater impairment after stroke [40], and interfering with the function of cPMd reduces behavioral performance to a greater extent among patients with greater poststroke impairment [41] as compared to patients with lesser impairment [42]. The behavioral relevance of these observations in more impaired patients may be similar to greater recruitment of secondary motor areas observed in healthy subjects during performance of increasingly complex tasks [43]. Together, these findings suggest a model for patients with more severe deficits in the chronic phase of stroke whereby increases in connectivity, especially between ipsilesional M1 and the contralesional hemisphere, during a course of poststroke rehabilitation therapy are important for achieving treatment-induced motor gains.

Among patients in the *less impaired subgroup*, larger treatment-induced motor gains were associated with *decreased* motor system functional connectivity between iM1 and contralesional areas, particularly between iM1-cPMd. Previous activation studies have shown that, while recruitment of secondary motor areas such as PMd can support motor recovery after stroke, restitution of normal circuitry is a more successful strategy [44]. Therefore, a model is suggested for patients with less severe deficits poststroke such that decreased reliance on functional connectivity between M1 and secondary motor regions, particularly contralesionally, during a course of poststroke rehabilitation is useful for achieving the best motor gains.

The current findings expand upon prior studies that studied BOLD fMRI functional connectivity measures as biomarkers of treatment gains in the chronic stroke setting. Várkuti et al. [17] treated nine patients with chronic stroke and severe motor deficits (mean FM score 18) using a brain-computer interface combined with robot assistance and found increases in connectivity between bilateral motor cortices in proportion to treatment-induced behavioral gains, echoing current findings in the more impaired group. Bajaj et al. [18] treated 10 patients with chronic stroke and moderate-severe motor deficits (mean FM score 35) using motor imagery combined with physical therapy and found that degree of behavioral gains correlated with the extent of increase in several forms of intrahemispheric connectivity. Young et al. [19] treated nine patients with chronic stroke and moderate motor deficits (ARAT score 27) using a brain-computer interface combined with functional electrical stimulation and found both decreases and increases in functional connectivity, within and between hemispheres. These studies included patients with relatively severe deficits, and so the tendency to see increased connectivity in parallel with the extent of treatment gains is consistent with the findings in the current study's more impaired group, where a similar pattern of treatment-induced increases in connectivity in proportion to behavioral gains was found. The current study extends these findings by including the analysis of a less impaired group, where greater treatment-related gains were associated with reductions in connectivity.

The current findings have implications for therapies aiming to modulate the interhemispheric balance of excitation and inhibition after stroke. Brain stimulation techniques such as transcranial direct current stimulation (tDCS) are under study to improve motor function after stroke. One strategy is advocated to decrease excitability within cM1 [45], based on the model that stroke produces increased cM1 inhibition of iM1 [46]. Current findings suggest that this approach may be more beneficial in patients with milder baseline deficits and less useful in those with greatest deficits and that a study enrolling both mild and severe cases may show reduced effect sizes. This view is concordant with prior findings whereby dampening excitability in cM1 with tDCS improved arm motor control in patients with milder impairment and worsened control in those with more severe impairment after stroke [21].

The strengths of the current study include a direct comparison of multiple biomarker candidates, including those with demonstrated value as well as the experimental SWI-based measure. Biomarkers were studied across a treatment regimen that used a robotic device to deliver therapy in a highly standardized manner. A relatively heterogeneous stroke population was intentionally enrolled, addressing the concern that studies using functional imaging to examine restorative therapy effects preferentially enroll a narrow fraction of patients with milder impairments [11] and enabling analysis of biomarker performance in relation to a wide range of baseline impairment levels. Weaknesses include the sample size, reduced due to serial data being unavailable in a number of patients. Treatment gains while statistically significant were overall modest, emphasizing the need to develop predictors, which can improve patient stratification, as well as biomarkers, which can provide greater insights into treatment effects and so may be useful to optimize dosing of poststroke restorative therapeutics. In addition, a potential confounder in the current study, as with any motor system study in a neurologically impaired population, is the impact of intersubject differences in motor task performance during fMRI acquisition. While differences in the range of motion did not reach statistical significance in the current cohort, current observations nonetheless suggest differences in motor task performance as a function of impairment level, which may have contributed to the current findings. However, fMRI activation is not simply a function of the range of motion but also reflects movement strength, attention, planning, effort, and sensory feedback. These features were not measured during fMRI acquisition in the current study. Thus, the current study of fMRI across a wide range of motor deficits after stroke must be interpreted in light of variance in motor task performance, as well as intersubject differences in numerous other variables that can affect fMRI measurements.

## 5. Conclusions

The human stroke population is extremely heterogeneous. A number of promising restorative therapies are under study. Their efficacy may be best appreciated by judicious use of biomarkers [8–10]. As noted by Bradnam et al. [21], this task “is not a ‘one-size-fits-all' approach.” A crutch improving function in more impaired patients hinders those who are less impaired. The current findings may be useful for defining biomarkers for restorative therapies after stroke across this complex population.

## Figures and Tables

**Table 1:** Main inclusion and exclusion criteria.

*Inclusion criteria for study entry*
(i) Age ≥ 18 years (ii) Diagnosis of stroke 11–26 weeks prior (iii) Residual arm motor deficit (ARAT < 52 or 9-hole peg test score > 25% longer than with unaffected hand) (iv) Preserved voluntary movements in distal upper extremity (≥5-degree range of motion in affected index metacarpophalangeal joint or wrist)
*Exclusion criteria for study entry*
(i) Contraindication to MRI (ii) Severe cognitive impairment (iii) Concurrent diagnosis affecting arm/hand function (iv) Arm motor status not at stable plateau (>3-point difference on Fugl-Meyer scale between the two baseline assessments)
*Exclusion criteria applied at time of data analysis*
(i) Excessive head motion during fMRI (either pre- or posttreatment scan) (ii) Posttreatment scan could not be obtained (e.g., claustrophobia or patient lost to follow-up)

**Table 2:** Patient characteristics.

	All patients	More severe at baseline (FM ≤ 36)	Less severe at baseline (FM > 36)	*p*
*n*	18	9	9	
Age (years)	61 ± 10	60 ± 10	62 ± 11	0.79
Sex	3 F/15 M	2 F/7 M	1 F/ 8 M	0.62
Time poststroke (months)	4.1 ± 0.97	4.5 ± 0.84	3.7 ± 0.96	0.07
Side of stroke	10 L/8 R	5 L/4 R	5 L/4 R	1.0
Infarct volume (cc)	27 ± 45 (0.5–166)	24 ± 37 (0.5–116)	30 ± 37 (0.7–165)	0.85
Corticospinal tract integrity^∗^	0.39 ± 0.11	0.37 ± 0.12	0.41 ± 0.10	0.31
Mean baseline Fugl-Meyer (normal = 66)	39 [20–60]	30 [20–35]	47.5 [38–60]	0.0003
NIH stroke scale (normal = 0)	4 ± 1	3.7 ± 1	4.3 ± 0.9	0.18
Nottingham sensory scale (normal = 17)	13 ± 4.5	13 ± 5.4	14 ± 3.7	0.76
Mini-mental state exam (normal = 30)	28 ± 2.6	28 ± 1.8	28 ± 3.3	0.65
Geriatric depression scale (normal = 0)	3.2 ± 2.1	3.4 ± 1.4	2.9 ± 2.7	0.59

Values are the mean ± SD (range) or median [IQR]. ^∗^Measured as fractional anisotropy within ipsilesional cerebral peduncle.

**Table 3:** Biomarker candidates under study.

*Functional MRI measures of regional activation*
Magnitude of activation (beta contrast estimates) in iM1, cM1, iPMd, and cPMd
Volume of activation in iM1, cM1, iPMd, and cPMd
*Measures of functional connectivity*
Functional connectivity between iM1 and cM1
Functional connectivity between iM1 and cPMd
Functional connectivity between iM1 and iPMd
*DTI measure of white matter integrity within corticospinal tract*
Fractional anisotropy within ipsilesional cerebral peduncle
*Peri-infarct angiogenesis*
T2^∗^-weighted signal in infarct Rims of two different diameters

M1: primary motor cortex; PMd: dorsal premotor cortex; i: ipsilesional; c: contralesional.

## References

[1] Cramer S. C. (2008). Repairing the human brain after stroke. II. Restorative therapies. *Annals of Neurology*.

[2] Cramer S. C. (2010). Stratifying patients with stroke in trials that target brain repair. *Stroke*.

[3] Hakkennes S., Hill K. D., Brock K., Bernhardt J., Churilov L. (2013). Selection for inpatient rehabilitation after severe stroke: what factors influence rehabilitation assessor decision-making?. *Journal of Rehabilitation Medicine*.

[4] Katz R. (2004). Biomarkers and surrogate markers: an FDA perspective. *NeuroRx*.

[5] Fleming T., DeMets D. (1996). Surrogate end points in clinical trials: are we being misled?. *Annals of Internal Medicine*.

[6] Hosp J. A., Luft A. R. (2011). Cortical plasticity during motor learning and recovery after ischemic stroke. *Neural Plasticity*.

[7] Byblow W. D., Stinear C. M., Barber P. A., Petoe M. A., Ackerley S. J. (2015). Proportional recovery after stroke depends on corticomotor integrity. *Annals of Neurology*.

[8] Milot M. H., Cramer S. C. (2008). Biomarkers of recovery after stroke. *Current Opinion in Neurology*.

[9] Burke E., Cramer S. C. (2013). Biomarkers and predictors of restorative therapy effects after stroke. *Current Neurology and Neuroscience Reports*.

[10] Boyd L. A., Hayward K. S., Ward N. S. (2017). Biomarkers of stroke recovery: consensus-based core recommendations from the stroke recovery and rehabilitation roundtable. *International Journal of Stroke*.

[11] Hodics T., Cohen L. G., Cramer S. C. (2006). Functional imaging of intervention effects in stroke motor rehabilitation. *Archives of Physical Medicine and Rehabilitation*.

[12] Scholz J., Klein M. C., Behrens T. E. J., Johansen-Berg H. (2009). Training induces changes in white-matter architecture. *Nature Neuroscience*.

[13] Taubert M., Draganski B., Anwander A. (2010). Dynamic properties of human brain structure: learning-related changes in cortical areas and associated fiber connections. *The Journal of Neuroscience*.

[14] Ding G., Jiang Q., Li L. (2011). Longitudinal magnetic resonance imaging of sildenafil treatment of embolic stroke in aged rats. *Stroke*.

[15] Carey J. R., Kimberley T. J., Lewis S. M. (2002). Analysis of fMRI and finger tracking training in subjects with chronic stroke. *Brain*.

[16] Cramer S. C., Parrish T. B., Levy R. M. (2007). Predicting functional gains in a stroke trial. *Stroke*.

[17] Várkuti B., Guan C., Pan Y. (2013). Resting state changes in functional connectivity correlate with movement recovery for BCI and robot-assisted upper-extremity training after stroke. *Neurorehabilitation and Neural Repair*.

[18] Bajaj S., Butler A. J., Drake D., Dhamala M. (2015). Brain effective connectivity during motor-imagery and execution following stroke and rehabilitation. *NeuroImage: Clinical*.

[19] Young B. M., Nigogosyan Z., Remsik A. (2014). Changes in functional connectivity correlate with behavioral gains in stroke patients after therapy using a brain-computer interface device. *Frontiers in Neuroengineering*.

[20] Grefkes C., Fink G. R. (2014). Connectivity-based approaches in stroke and recovery of function. *The Lancet Neurology*.

[21] Bradnam L. V., Stinear C. M., Barber P. A., Byblow W. D. (2012). Contralesional hemisphere control of the proximal paretic upper limb following stroke. *Cerebral Cortex*.

[22] See J., Dodakian L., Chou C. (2013). A standardized approach to the Fugl-Meyer assessment and its implications for clinical trials. *Neurorehabilitation and Neural Repair*.

[23] Takahashi C. D., Der-Yeghiaian L., Le V., Motiwala R. R., Cramer S. C. (2008). Robot-based hand motor therapy after stroke. *Brain*.

[24] Yozbatiran N., Der-Yeghiaian L., Cramer S. C. (2008). A standardized approach to performing the action research arm test. *Neurorehabilitation and Neural Repair*.

[25] Ward N. S., Brown M. M., Thompson A. J., Frackowiak R. S. J. (2003). Neural correlates of outcome after stroke: a cross-sectional fMRI study. *Brain*.

[26] Burke Quinlan E., Dodakian L., See J. (2015). Neural function, injury, and stroke subtype predict treatment gains after stroke. *Annals of Neurology*.

[27] Burke E., Dodakian L., See J. (2014). A multimodal approach to understanding motor impairment and disability after stroke. *Journal of Neurology*.

[28] O’Reilly J. X., Woolrich M. W., Behrens T. E. J., Smith S. M., Johansen-Berg H. (2012). Tools of the trade: psychophysiological interactions and functional connectivity. *Social Cognitive and Affective Neuroscience*.

[29] Saleh S., Adamovich S. V., Tunik E. Resting state functional connectivity and task-related effective connectivity changes after upper extremity rehabilitation: a pilot study.

[30] Kim J., Horwitz B. (2008). Investigating the neural basis for fMRI-based functional connectivity in a blocked design: application to interregional correlations and psycho-physiological interactions. *Magnetic Resonance Imaging*.

[31] Stewart J. C., Tran X., Cramer S. C. (2014). Age-related variability in performance of a motor action selection task is related to differences in brain function and structure among older adults. *NeuroImage*.

[32] van Meer M. P. A., van der Marel K., Wang K. (2010). Recovery of sensorimotor function after experimental stroke correlates with restoration of resting-state interhemispheric functional connectivity. *The Journal of Neuroscience*.

[33] Cramer S. C., Shah R., Juranek J., Crafton K. R., Le V. (2006). Activity in the peri-infarct rim in relation to recovery from stroke. *Stroke*.

[34] Woytowicz E. J., Rietschel J. C., Goodman R. N. (2017). Determining levels of upper extremity movement impairment by applying a cluster analysis to the Fugl-Meyer assessment of the upper extremity in chronic stroke. *Archives of Physical Medicine and Rehabilitation*.

[35] Carter A. R., Patel K. R., Astafiev S. V. (2012). Upstream dysfunction of somatomotor functional connectivity after corticospinal damage in stroke. *Neurorehabilitation and Neural Repair*.

[36] Carter A. R., Shulman G. L., Corbetta M. (2012). Why use a connectivity-based approach to study stroke and recovery of function?. *NeuroImage*.

[37] Carter A. R., Astafiev S. V., Lang C. E. (2010). Resting interhemispheric functional magnetic resonance imaging connectivity predicts performance after stroke. *Annals of Neurology*.

[38] Sergi F., Krebs H. I., Groissier B. Predicting efficacy of robot-aided rehabilitation in chronic stroke patients using an MRI-compatible robotic device.

[39] Duncan P. W., Lai S. M., Keighley J. (2000). Defining post-stroke recovery: implications for design and interpretation of drug trials. *Neuropharmacology*.

[40] Bestmann S., Swayne O., Blankenburg F. (2010). The role of contralesional dorsal premotor cortex after stroke as studied with concurrent TMS-fMRI. *The Journal of Neuroscience*.

[41] Johansen-Berg H., Rushworth M. F. S., Bogdanovic M. D., Kischka U., Wimalaratna S., Matthews P. M. (2002). The role of ipsilateral premotor cortex in hand movement after stroke. *Proceedings of the National Academy of Sciences of the United States of America*.

[42] Fridman E. A., Hanakawa T., Chung M., Hummel F., Leiguarda R., Cohen L. (2004). Reorganization of the human ipsilesional premotor cortex after stroke. *Brain*.

[43] Rao S. M., Binder J. R., Bandettini P. A. (1993). Functional magnetic resonance imaging of complex human movements. *Neurology*.

[44] Ward N., Brown M., Thompson A., Frackowiak R. (2003). Neural correlates of motor recovery after stroke: a longitudinal fMRI study. *Brain*.

[45] Hummel F. C., Cohen L. G. (2006). Non-invasive brain stimulation: a new strategy to improve neurorehabilitation after stroke?. *The Lancet Neurology*.

[46] Murase N., Duque J., Mazzocchio R., Cohen L. G. (2004). Influence of interhemispheric interactions on motor function in chronic stroke. *Annals of Neurology*.

